# Modulating macrophage function to reinforce host innate resistance against *Mycobacterium avium* complex infection

**DOI:** 10.3389/fimmu.2022.931876

**Published:** 2022-11-24

**Authors:** Hyun-Eui Park, Wonsik Lee, Sangwon Choi, Myunghwan Jung, Min-Kyoung Shin, Sung Jae Shin

**Affiliations:** ^1^ Department of Microbiology and Convergence Medical Science, Institute of Health Sciences, College of Medicine, Gyeongsang National University, Jinju, South Korea; ^2^ School of Pharmacy, Sungkyunkwan University, Suwon, South Korea; ^3^ Department of Microbiology, Institute for Immunology and Immunological Disease, Graduate School of Medical Science, Brain Korea 21 Project, Yonsei University College of Medicine, Seoul, South Korea

**Keywords:** nontuberculous mycobacteria, *Mycobacterium avium* complex, innate immunity, macrophage, host-directed therapy

## Abstract

*Mycobacterium avium* complex (MAC) is the main causative agent of infectious diseases in humans among nontuberculous mycobacteria (NTM) that are ubiquitous organisms found in environmental media such as soil as well as in domestic and natural waters. MAC is a primary causative agent of NTM-lung disease that threaten immunocompromised or structural lung disease patients. The incidence and the prevalence of *M. tuberculosis* infection have been reduced, while MAC infections and mortality rates have increased, making it a cause of global health concern. The emergence of drug resistance and the side effects of long-term drug use have led to a poor outcome of treatment regimens against MAC infections. Therefore, the development of host-directed therapy (HDT) has recently gained interest, aiming to accelerate mycobacterial clearance and reversing lung damage by employing the immune system using a novel adjuvant strategy to improve the clinical outcome of MAC infection. Therefore, in this review, we discuss the innate immune responses that contribute to MAC infection focusing on macrophages, chief innate immune cells, and host susceptibility factors in patients. We also discuss potential HDTs that can act on the signaling pathway of macrophages, thereby contributing to antimycobacterial activity as a part of the innate immune response during MAC infection. Furthermore, this review provides new insights into MAC infection control that modulates and enhances macrophage function, promoting host antimicrobial activity in response to potential HDTs and thus presenting a deeper understanding of the interactions between macrophages and MACs during infection.

## Introduction

Nontuberculous mycobacteria (NTM) are opportunistic pathogens widely distributed in environment such as soil and water (1). They are opportunistic pathogens. The *Mycobacterium avium* complex (MAC) of NTM is the main cause of infection and disease in humans (1). MAC is considered as an opportunistic pathogen in populations predisposed to immunosuppression contributed by human immunodeficiency virus infection, cystic fibrosis, chronic obstructive pulmonary disease, bronchiectasis, and long-term use of immunosuppressants (2). Recent findings suggest its role in causing chronic pulmonary disease in immunocompetent adults (3–5).

NTM infections, including those caused by MAC, are divided into four clinical types—pulmonary disease, lymphadenitis, skin/cellulitis, and disseminated disease (6). In particular, pulmonary infection in immunocompromised patients with an underlying pulmonary disorder and disseminated disease is the foremost clinical syndrome caused by MAC infection (2, 6, 7). Disseminated disease caused by MAC occurs when the T cell-mediated immune response is affected in cases of a high dose of immunosuppressants, idiopathic CD4^+^ lymphocytopenia, and advanced stage acquired immunodeficiency syndrome. In immunocompetent hosts, local infection mainly occurs in the respiratory tract. In chronic infections accompanied by colonization and inflammation of the respiratory system, disturbance of the innate immune system is the main factor.

MAC can trigger anti-inflammatory cytokines, inhibit key innate immune functions, and manipulate the complex immune signaling pathways of the host, thereby allowing bacterial growth within host macrophages. Therefore, in this review, to understand the pathogenesis and host defense mechanism of MAC, in addition to the immune responses common to mycobacterial infections, including tuberculosis (TB), the characteristic macrophage signaling pathway of MAC that can contribute to pathogenesis is explained. We aim to understand the pathogenesis and control mechanisms of MAC by modulating host susceptibility factors related to innate immunity.

Thus far, the treatment of MAC pulmonary disease has remained challenging. It relies on a three-drug regimen— rifamycin, ethambutol, and macrolide—for at least 12 months after negative sputum culture (8). Even after the completion of the course of medications recommended by the American Thoracic Society, the success rate of treatment is as low as 66% (9). MAC is highly resistant to primary antimicrobial agents because of features such as impermeable cell walls, mutations in drug target genes, drug efflux pumps, and biofilm formation (10). In addition, *in vitro* drug susceptibility testing and *in vivo* clinical outcomes show a poor correlation, except for macrolide and amikacin in MAC infection (6). Furthermore, the long-term use of several antibiotics causes side effects such as diarrhea, gastrointestinal disorders, hearing loss, and hepatitis, in which cases the treatment is often terminated, thus hindering MAC control (11). Therefore, new approaches for treating MAC pulmonary disease with improved therapeutic response and shortened treatment duration are needed.

Host-directed therapy (HDT) targets host immunity rather than directly attacking pathogens. HDT boosts the protective immune response and rehabilitates inhibited or modulated immune responses by the pathogen. In addition, it may eliminate non-replicating antibiotic-tolerant persisters that survive antibiotic therapy. Finally, HDT has several potential advantages, including a reduced treatment period, immunopathological tissue damage, and adverse drug effects. Therefore, herein we discuss the major innate immune response in MAC infection with particular emphasis on the macrophage signaling pathway. Accordingly, an improved HDT-based treatment for MAC infections is discussed.

## Understanding the innate immune response to MAC infection

The host’s innate immune response plays a major role in chronic MAC infection, accompanied by MAC colonization and inflammation in the lungs. Therefore, to understand the pathogenesis and control mechanism of MAC, understanding the host’s innate immune response is important. This chapter summarizes the innate immune response to MAC infection, focusing on signaling pathways in macrophages, the innate immune cells post-infection, and acquired and congenital host susceptibility factors related to innate immunity in the patients.

### Overview of the immune response in MAC-infected macrophage

**Figure 1:**
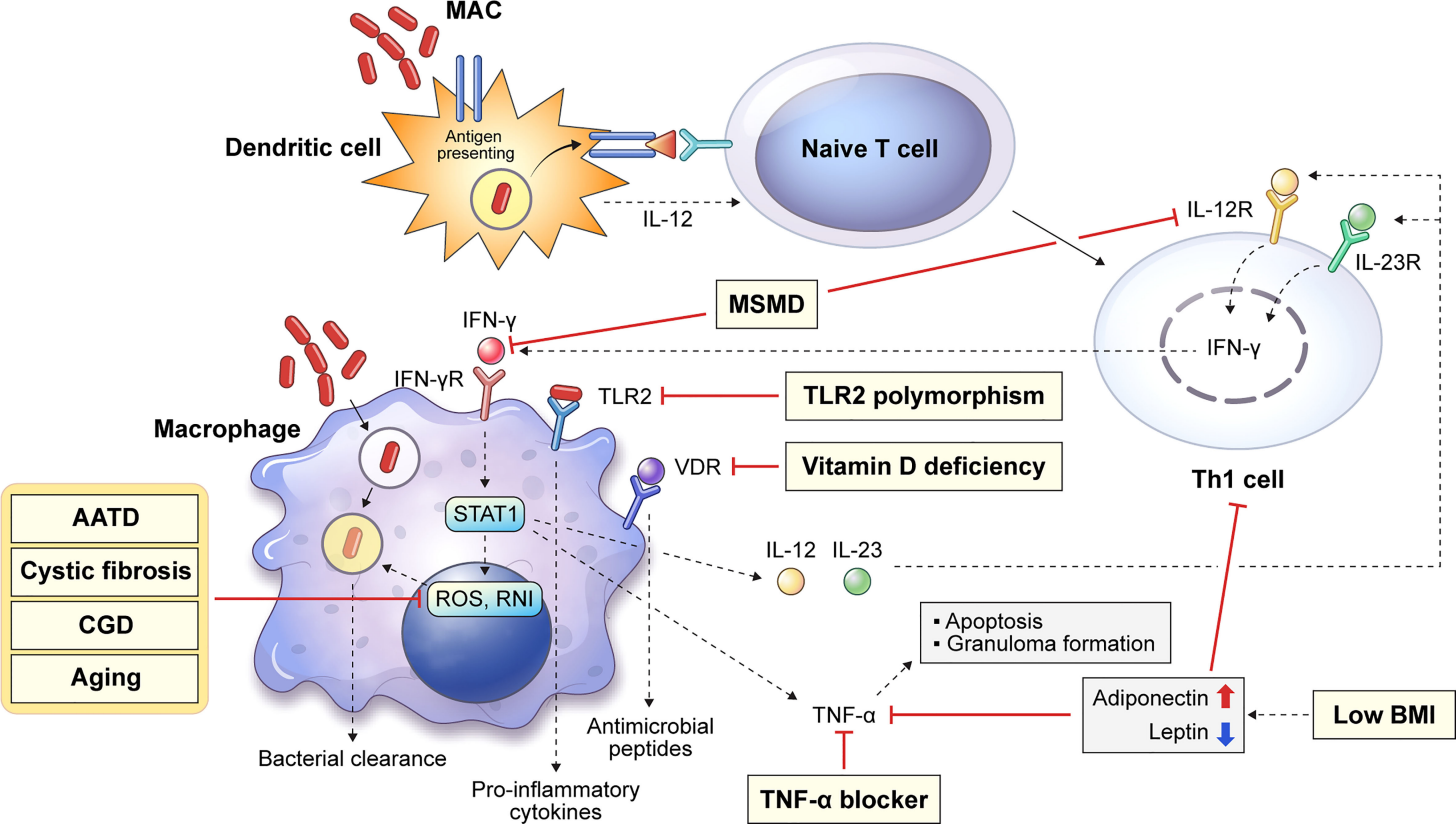
Overview of representative host innate genetic defects increasing the susceptibility to MAC infection. Host susceptibility factors to MAC infection can infer important innate immunological functions against the infection in the host. Several innate or acquired host factors can increase the susceptibility to MAC infection by suppressing the host defense, and these factors are shown in the corresponding signaling pathways (yellow box). Dendritic cells endocytose mycobacteria, which induces the presentation of antigens to naïve T cells. Antigen presentation induces the differentiation of naïve T cells into Th1 cells causing macrophage activation, which produces reactive oxygen and nitrogen species and leads to intracellular bacterial death. In addition, IFN-γ and TLR2 signaling induces the production of pro-inflammatory cytokines, such as IL-12, IL-23, and TNF-α. Secreted IL-12 and IL-23 bind to their receptors on Th1 cells and enhance IFN-γ production. TNF-α production promotes macrophage apoptosis and granuloma formation. MAC, *Mycobacterium avium* complex; MSMD, Mendelian susceptibility to mycobacterial disease; AATD, alpha-1 antitrypsin deficiency; CGD, chronic granulomatous disease; BMI, body mass index; IFN, interferon; TLR, Toll-like receptor; IL, interleukin; TNF, tumor necrosis factor.

The major innate immune cells involved in MAC infection are macrophages and dendritic cells (DCs) that engulf and eliminate invading pathogens. **Figure 1** shows a schematic diagram of the host innate defense mechanism or bacterial survival due to MAC infection by the major innate immune cells. DCs are antigen-presenting cells that link innate and adaptive immunity. They play an important role in killing of intracellular mycobacteria by presenting antigens to naïve T cells and inducing T cell responses (12, 13). Antigen presentation induces the differentiation of naïve T cells into Th1 cells, which leads to macrophage activation (14). Macrophages can be activated by a specific signaling cascade when infected with MAC or by Th1 cells induced by DCs (14). IFN-γ and Toll-like receptor 2 (TLR2) signaling induces the production of pro-inflammatory cytokines, such as interleukin (IL)-12, IL-23, and tumor necrosis factor (TNF)-α. Secreted IL-12 and IL-23 bind to their receptors on Th1 cells and enhance IFN-γ production. Secreted TNF-α may induce bacterial clearance by apoptosis or participate in granuloma formation in the lungs (**Figure 1**). Several congenital or acquired host factors may inhibit the host defense response, leading to an increased susceptibility to MAC infection. Host susceptibility factors to MAC infection, such as Mendelian susceptibility to mycobacterial disease (MSMD), alpha-1 antitrypsin (AAT) deficiency, cystic fibrosis, chronic granulomatous disease, and aging, which affect macrophage function, are indicated by the corresponding signaling cascade in macrophages (**Figure 1**). Detailed descriptions are provided in this chapter.

**Figure 2:**
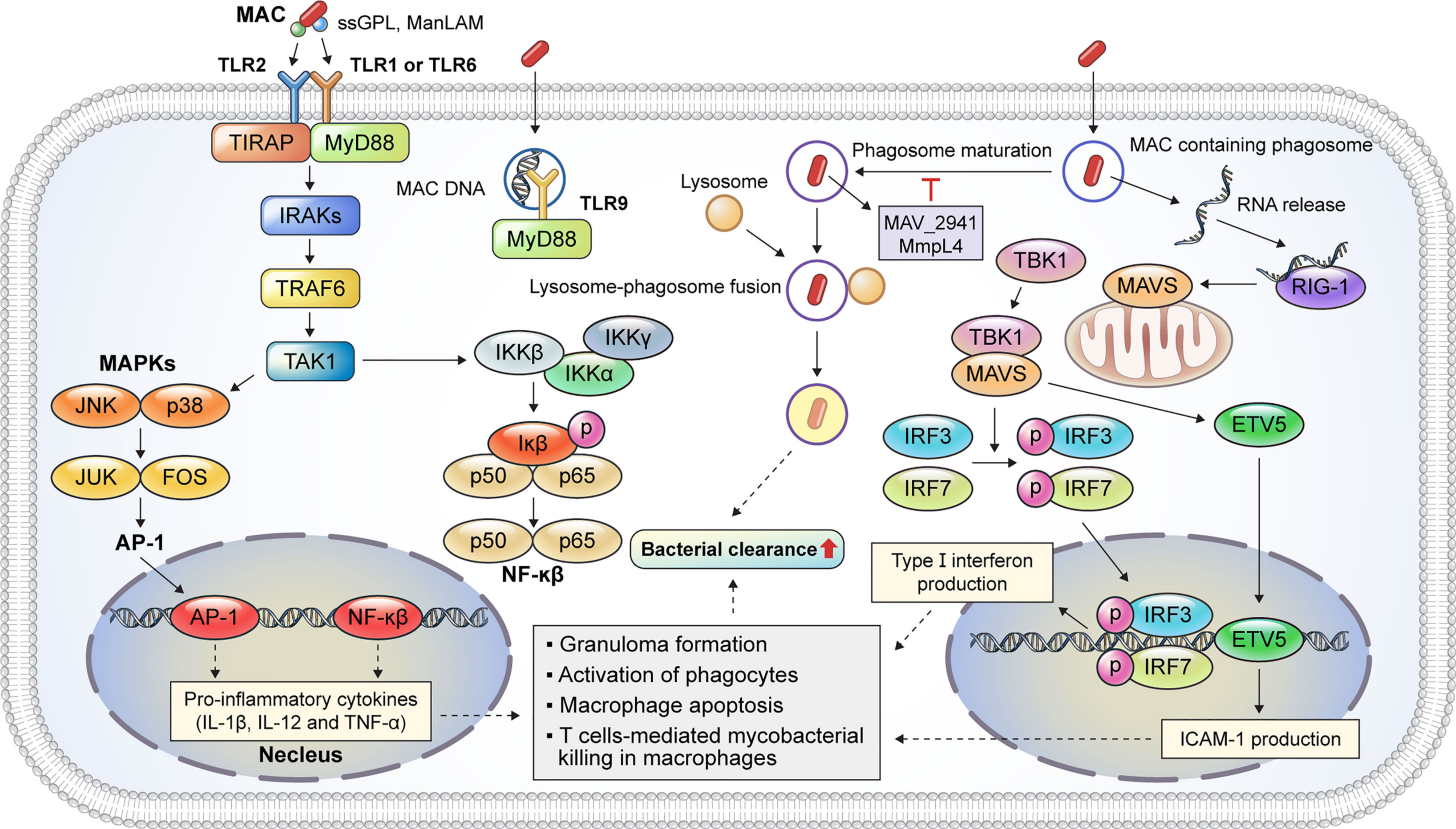
Representative host protective innate immune mechanisms of macrophages against MAC infection. Macrophages are immune cells that play a key role in host innate defense mechanisms against MAC infection. TLR2 signaling is activated by the binding of mycobacterial PAMP, such as ssGPL and ManLAM, to TLR heterodimers, which induces MyD88-dependent cascade that activates the kinase activity of the IRAK complex. TLR9 recognizes MAC DNA and promotes a MyD88-dependent response. The IRAK complex activates TRAF6, which, in turn, activates TAK-1. Activation of TAK-1 leads to the translocation of AP-1 and NF-κβ into the nucleus to produce pro-inflammatory cytokines, such as IL-1β, IL-12, and TNF-α. These cytokines augment the antimycobacterial response, including granuloma formation, phagocyte activation, and apoptosis. MAC-containing phagosomes undergo maturation and fusion events to eliminate the phagocytosed bacteria. *M. avium* proteins such as MAV_2941 and MMPL4 inhibit phagosome maturation, which subsequently promotes intracellular survival. The recognition of bacterial RNA plays a critical role in the innate immune response during MAC infection. The released MAC RNA binds to RIG-I, which induces a MAVS-dependent response, thereby activating TBK-1. Activated TBK-1 and MAVS promote the nuclear translocation of IRF3/7 and ETV5, leading to type I interferon and ICAM-1 expression, respectively. Taken together, the innate immune response induced by TLR2/1, TLR2/6, and TLR9 signaling and host cytosolic RNA-sensing pathways contributes to bacterial clearance during MAC infection. MAC, *Mycobacterium avium* complex; TLR, Toll-like receptor; PAMP, pathogen-associated molecular pattern; ssGPL, species-specific glycopeptidolipid; ManLAM, mannose-capped lipoarabinomannan; IRAK, interleukin-1 receptor-associated kinase; TRAF6, tumor necrosis factor receptor (TNFR)-associated factor 6; TAK-1, transforming growth factor-β (TGF-β)-activated kinase 1; AP-1, activator protein 1; NF, nuclear factor; IL, interleukin; TNF, tumor necrosis factor; RIG-I, retinoic acid inducible gene-I; MAVS, mitochondrial antiviral signaling protein; TBK-1, TANK-binding kinase-1; IRF, interferon regulatory factor; ETV, Ets variant; ICAM, intracellular adhesion molecule.

Macrophages play a critical role in MAC infection, ranging from pathogen recognition to the elimination of internalized mycobacteria. The inhalation of aerosols containing MAC from environmental sources initiates a pulmonary MAC infection (15) where alveolar macrophages are the primary site of infection (6). Thus, MAC is directly recognized by macrophages; the immune response caused by an infection is summarized in **Figure 2**. Pathogen recognition during MAC infection begins with interactions between alveolar macrophage pattern recognition receptors (PRRs) and pathogen-associated molecular patterns (PAMPs) (15). Mycobacterial cell wall components, such as mycolic acid, lipomannan, lipoarabinomannan, and phosphatidylinositol mannosides, are representative ligands for PRRs (15). TLRs and C-type lectins are representative PRRs that recognize bacterial PAMPs. The activation of TLRs induces the signal transduction pathway, which recruits downstream signaling molecules, including MyD88, IRAK1, IRAK4, TRAF6, TAK1, TAB1, and TAB2 (16). In addition, TLR activation induces the production of reactive oxygen species (ROS) and reactive nitrogen species for the direct killing of internalized mycobacteria (17, 18). Previous studies have revealed that TLRs play a pivotal role in the initiation of pro-inflammatory cytokine production and signal transduction pathways during MAC infection (**Figure 2**). *M. avium* infection induces the upregulation of TLR2 expression in murine macrophages (19). TLR2 activation provides host protection against MAC infection *via* the induction of Th1 cytokines, such as IL-12p40 and TNF-α, *in vitro* and *in vivo* (19, 20). Furthermore, according to Feng et al., TLR2- and MyD88-deficient mice showed a higher bacterial burden and increased susceptibility to MAC infection than wild-type mice (21). In contrast, TLR9 deficiency did not affect *M. avium* growth in macrophages (22). However, TLR9, TLR2, or MyD88 deficiency was found to induce a decreased number of macrophages, epithelioid cells, and lymphocytes within granulomas in *M. avium*-infected mice (22). In addition, TLR2 expression was lower in patients with NTM lung disease than in healthy controls, and the secretion of IL-12p40 and TNF-α was reduced in both unstimulated and *M. avium*-stimulated monocytes isolated from patients with NTM lung disease (23).

After recognition, membrane protrusions surround and phagocytose the pathogen (24). The newly produced phagosomes interact with endocytic organelles for phagosome maturation (24). Phagosomes then combine with lysosomes containing antimicrobial peptides and degrading enzymes to form phagolysosomes for the intracellular killing of the pathogen (25). However, MAC can resist host defense mechanisms and persist in macrophages—for example, the secretory protein MAV_2941 inhibits phagosome maturation by interfering with lysosomal-associated membrane protein 1 (26). Moreover, voltage-dependent anion channels (VDACs) are involved in the inhibition of phagosome maturation in *M. avium*-infected macrophages (27). VDACs were co-localized with the mycobacterial membrane protein MMPL4, and VDAC inhibition significantly reduced the viability of *M. avium* in macrophages (27).

Apoptosis of macrophages leads to the clearance of intracellular bacteria, suggesting that apoptosis provides host protection against MAC infection (28, 29). However, another study suggested that *M. avium* escapes from apoptotic macrophages and infects new macrophages as the infection progresses (30). In addition, *M. avium* protein MAV_2504 promotes macrophage apoptosis by inducing mitochondrial damage and cytochrome c release (31). Furthermore, Agdestein et al. reported that *M. avium* clinical strains induced apoptosis in human peripheral blood mononuclear cells (PBMCs) (32). Conversely, *M. avium* suppressed apoptosis signaling in canine PBMCs, indicating that it differentially modulates apoptosis in macrophages depending on the host species (33).

Host cytosolic RNA-sensing pathways play a critical role in sensing the DNA and RNA of intracellular pathogens, including *Listeria monocytogenes* and *M. tuberculosis* (Mtb) (34, 35). The host cytosolic RNA-sensing pathway facilitates T cell-mediated bacterial killing in *M. avium*-infected macrophages (36). In macrophages, RNA released from MAC-containing phagosomes and retinoic acid inducible gene-I (RIG-I) recognizes the released RNA (**Figure 2**). RIG-I interacts with mitochondrial antiviral signaling protein (MAVS) and recruits TANK-binding kinase-1 (TBK-1). The binding of TBK-1 to MAVS promotes the phosphorylation of interferon regulatory factor 3 (IRF3) and interferon regulatory factor 5 (IRF5). The phosphorylated IRF3 and IRF5 translocate to the nucleus and promote the production of type I interferon (**Figure 2**). In addition, TBK-1 and MAVS promote the nuclear translocation of Ets variant 5 (ETV5). The nuclear translocation of ETV5 induces the expression of intracellular adhesion molecule-1 (ICAM-1) which, along with type I interferon expression, induces T lymphocyte-mediated mycobacterial killing in macrophages. In contrast to Mtb infection, activation of the host RNA-sensing pathway provides host protection against *M. avium* infection (36).

Therefore, TLR and host cytosolic RNA-sensing pathways play a central role in the innate immune response of macrophages during MAC infection. Although macrophages act as the first line of defense in MAC infection, they also provide an intracellular replication niche for MAC to support the process of dispersal. Currently, the interaction between macrophages and MAC is less understood than that between macrophages and Mtb, particularly at the molecular level. Therefore, understanding macrophage–MAC interactions is critical for developing HDTs to improve the clinical outcome of MAC infection.

### Host susceptibility factors of MAC infection involved in macrophage functions

The host susceptibility factors for MAC infection are broadly divided into structural lung abnormalities and immune dysfunction (37). Congenital factors include cystic fibrosis, primary ciliary dyskinesia, AAT deficiency, pulmonary alveolar proteinosis, chronic granulomatous disease, TLR2 polymorphism, and MSMD (37, 38). Acquired factors include emphysema, bronchiectasis, vitamin D deficiency, silicosis, and administration of immunosuppressive drugs, such as corticosteroids and TNF-α antagonists (39–41). In this chapter, we summarize the host susceptibility factors related to MAC infection reported to date and facilitate the inference of the important immunological function for MAC infection in the host. The congenital and acquired factors involved in MAC infection, associated host genes, relevance to macrophage functions, and biological effects are listed in **Table 1**.

**Table 1:** Primary and acquired host susceptibility factors associated with *Mycobacterium avium* complex infection and their involvement in macrophage function.

Host susceptibility factors	Associated host genes	Relevance to macrophage functions	Biological effect	References
MSMD	*IL-12p40*, *IL-12Rβ1*, *TYK2*, *SPPL2a*, and *ISG15*	Decreased production of IFN-γ	Increased susceptibility to disseminated nontuberculous mycobacteria infection due to the impaired bacterial clearance	(38, 42, 43)
	*IFN-γR1*, *IFN-γR2*, *STAT1*, and *gp91phox*	Impaired response to IFN-γ		
	*IRF8* and *NEMO*	Decreased production of IFN-γ Impaired response to IFN-γ	
Cystic fibrosis	*CFTR*	Downregulated phagocytic surface marker Overexpression of pro-inflammatory cytokines and downregulation of anti-inflammatory cytokines	Impaired bacterial clearance and worsened lung immunopathology	(44–47)
Alpha-1-antitrypsin deficiency	*SERPINA1*	Misfolding of alpha-1 antitrypsin deficiency induces the overexpression of pro-inflammatory cytokines and proteolysis	Worsened lung immunopathology	(48–50)
Chronic granulomatous disease	*gp91^phox^* *p22^phox^ p47^phox^ p67^phox^*	Mutation of NADPH oxidase complex leads to impaired intracellular killing	Higher bacterial burden and mortality	(51–55)
Tumor necrosis factor-α blockers and immunosuppressive drugs	N/A	Downregulation of pro-inflammatory cytokines and upregulation of anti-inflammatory phenotype	Resolution of local inflammation Impaired bacterial clearance with prolonged usage	(39, 56–62)
Vitamin D deficiency	*VDR*	Impaired macrophage activation Decreased production of cathelicidin	Impaired bacterial clearance	(63–66)
TLR2 polymorphism	*TLR2*	Downregulation of pro-inflammatory cytokines	Impaired bacterial clearance	(67, 68)
Low body mass index	Unknown	Promotion of M2 macrophage polarization and anti-inflammatory cytokines Suppression of M1 macrophage polarization and pro-inflammatory cytokines	Impaired bacterial clearance due to anti-inflammatory response	(69–72)
Aging	Unknown	Depressed expression of pro-inflammatory cytokines Promotion of M2 macrophage polarization Impaired phagocytosis	Impaired bacterial clearance due to anti-inflammatory response	(73–79)

N/A, not applicable.

#### MSMD

MSMD is a genetic disorder that shows high susceptibility to infection with low-virulence mycobacteria, such as *M. bovis* and NTM. Patients with MSMD are highly susceptible to various pathogens, including *Salmonella*, *Candida*, *Histoplasma*, *Coccidioides*, and other bacteria and viruses (38). To date, mutations in 11 genes have been identified to cause MSMD (42). Of these, five (IL-12p40, IL-12Rβ1, TYK2, *SPPL2a*, and ISG15) are associated with the production of IFN-γ, and four (IFN-γR1, IFN-γR2, STAT1, and *gp91^phox^*) are involved in the response to IFN-γ (42) (**Table 1**). The remaining two genes (*IRF8* and NEMO) are involved in both the production and response of IFN-γ (42). Innate immunity to mycobacterial infection requires delicate interactions between lymphoid cells and macrophages. Macrophages secrete IL-12 after the phagocytosis of mycobacteria to stimulate T cells and natural killer cells *via* the IL-12 receptor composed of a heterodimer of IL12RB1 and IL12RB2 (38). IL-12 receptor signaling *via* TYK2 and JAK2 induces STAT4 phosphorylation, homodimerization, and translocation to the nucleus, leading to IFN-γ production (38). The binding of IFN-γ to IFNGR, which is composed of heterodimers of IFNGR1 and IFNGR2, leads to the phosphorylation of JAK1, JAK2, STAT1, and phosphorylated STAT1 (pSTAT1) homodimerization. Subsequently, pSTAT1 translocates to the nucleus and leads to the upregulation of IFN-γ-responsive gene transcription binding with IFN-γ activation sequence elements (38). These transcriptional changes induce an antimycobacterial response, including macrophage activation and the expression of pro-inflammatory cytokines such as IL-12 and TNF-α (38). Finally, activated macrophages facilitate the elimination of intracellular mycobacteria by phagosome acidification and lysosomal delivery, nutrient starvation, autophagy, and the production of antimicrobial peptides and ROS (38). Mutations in *IFNGR1* and *IL12RB1* are the most common causes of MSMD, accounting for 39% and 41% of genetically diagnosed cases, respectively (43). These mutations induce an impaired response to IFN-γ, thus increasing the susceptibility to disseminated NTM infections. Taken together, mutations in genes encoding receptors and cytokines of the signal transduction pathways in the IL-12/IFN-γ axis induce a high susceptibility to MAC infection in both pulmonary and extrapulmonary diseases.

#### Cystic fibrosis

Cystic fibrosis (CF) is a lethal genetic disorder caused by mutations in the cystic fibrosis transmembrane conductance regulator (CFTR) gene, which encodes a chloride channel protein (80). CFTR dysfunction induces abnormal ion transport, promoting a diminished mucus layer in the airway (80). A decreased mucus layer leads to impaired ciliary clearance, thus increasing the susceptibility to bacterial colonization and infection (81). Previous studies have shown that macrophages isolated from patients with CF exhibit distinct phenotypes (44–47). Several surface markers involved in complement-mediated phagocytosis, such as urokinase plasminogen-activator receptor and CD11b, are downregulated in monocytes from CF patients (44). Additionally, macrophages from the sputum of CF patients have a low expression of scavenger receptors such as MARCO and CD206 (45). The CF macrophages also display a reduced expression of caveolin-1, which is involved in phagocytosis and intracellular killing of *Pseudomonas aeruginosa* (46). A reduced expression of caveolin-1 leads to hyperinflammation, which is caused by intracellularly localized heme oxygenase-1 (HO-1) (47). The levels of pro-inflammatory cytokines, such as TNF-α, IL-1β, and IL-6, are higher in the lungs of patients with CF than in healthy controls (82, 83). In contrast, the level of IL-10, which promotes the resolution of inflammation, is reduced in bronchoalveolar lavage fluid and macrophages (82). CF macrophages induce hyperinflammation, resulting in lung tissue injury. Upregulated pro-inflammatory responses and reduced resolution may promote local inflammation and immunopathology of the lungs of CF patients. These results suggest that macrophages from patients with CF show a hindered phagocytic activity, leading to impaired bacterial clearance (**Table 1**). Furthermore, the downregulation of surface markers involved in phagocytosis and excessive inflammation in CF patients may induce delayed bacterial clearance and lung tissue injury.

#### AAT deficiency

AAT plays a role in protecting various organs by inactivating proteinases, including neutrophil elastase (NE), cathepsin G, and proteinase, for tissue homeostasis (84). AAT deficiency (AATD) is an autosomal codominant genetic disorder with over 120 reported genotypes (84). Mutation of the SERPINA1 gene induces the accumulation of misfolded ATT proteins in hepatocytes, promoting insufficient circulating AAT (83). AATD shows a relatively low serum concentration of AAT (2.5–7 µmol/L) compared with healthy control (20–48 µmol/L) (84). In AAT-deficient patients, NE degrades lung tissue, resulting in early-onset emphysema and liver disease (85). Macrophages contribute to AAT production in the lungs and prevent tissue damage from proteinases (86). Under healthy conditions, monocytes release AAT extracellularly (86). AAT blocks lipopolysaccharide (LPS)-mediated pro-inflammatory cytokine production, thereby preventing excessive inflammation in local tissue (86). However, in AATD, the production of AAT polymer due to misfolding leads to the upregulation of pro-inflammatory cytokines such as CXCL1, CXCL2, TNF-α, IL-1, IL-6, and IL-8, thereby promoting neutrophil recruitment (86, 87). Therefore, AATD induces excessive inflammation and subsequent lung tissue injury (**Table 1**). Krotova et al. identified that macrophages from AATD show elevated levels of the membrane-bound serine protease matriptase, which promotes the proteolysis of the extracellular matrix (ECM) (48). In addition, upregulation of matriptase induces the activation of MMP-14, thus promoting ECM degradation (48). Tissue damage due to excessive pro-inflammatory cytokines and proteolysis in AATD may be a predisposing factor for NTM lung disease. In addition, AAT enhances macrophage function in response to NTM infection. AAT-treated macrophages show reduced bacterial burden with enhanced phagosome–lysosome fusion and autophagosome formation/maturation in *M. intracellulare* infection (49). In addition, AAT and serine proteinase inhibitors reduce the bacterial burden of *M. abscessus* infection in macrophages (50). Thus, AAT provides host protection against NTM infection by enhancing macrophage function and limiting tissue damage caused by excessive inflammation (**Table 1**).

#### Chronic granulomatous disease

Chronic granulomatous disease (CGD) is a rare genetic disorder that affects the activity of the nicotinamide adenine dinucleotide phosphate (NADPH) oxidase complex of phagocytic cells (88). The NADPH oxidase complex facilitates the transfer of electrons from NADPH to oxygen, inducing superoxide production for antimicrobial activity (89). The NADPH oxidase complex consists of several subunits, including the membrane and cytosolic compartments (89). The membrane compartment is a cytochrome-b558 heterodimer which consists of gp91^phox^ and p22^phox^ (89). The cytosolic compartment consists of p67^phox^, p47^phox^, and p40^phox^ (89). Mutations in the subunit of the NADPH oxidase complex impair enzyme activity and thus suppress the respiratory burst in phagocytes (88). X-linked CGD due to mutation in the *CYBB* gene encoding gp91^phox^ accounts for 65% of the diagnosed CGD cases in Europe and North America (90, 91). Autosomal recessive CGD due to mutations in other components of the NADPH oxidase complex is responsible for the remaining 35% of the cases (89). Of these, the mutations in p22^phox^, p47^phox^, and p67^phox^ account for 25%, 5%, and 5% of the cases, respectively (89). CGD leads to the suppression of respiratory bursts in phagocytes, resulting in impaired bacterial clearance (91, 92) (**Table 1**). Patients with CGD are highly susceptible to mycobacterial infections, including Mtb and *M. bovis* (51). Furthermore, several studies have reported NTM infections in CGD patients (52–54). CGD mice showed a higher bacterial burden and mortality rate than wild-type mice after an intratracheal infection with *M. avium* (55). In addition, macrophages from CGD mice had impaired intracellular killing ability compared with those from wild-type mice (55). These observations suggest that defects in NADPH oxidase function induce high susceptibility to NTM infection.

#### TNF-α blockers and immunosuppressive drugs

TNF-α is a pro-inflammatory cytokine that promotes monocyte–macrophage differentiation, phagosome maturation, and neutrophil recruitment during infection (93, 94). The TNF-α pathway is an important therapeutic target for autoimmune diseases, such as rheumatoid arthritis, psoriasis, seronegative spondyloarthropathies, and inflammatory bowel disease (93). To date, five TNF-α blockers, namely, infliximab, adalimumab, certolizumab pegol, golimumab, and etanercept, have been approved and used for the treatment of autoimmune diseases (95). However, the use of TNF-α blockers significantly increases the risk of Mtb infection. The rate of TB is higher in patients who received TNF-α blocker treatment (54, 56, 89, 91, 94, 96–98). Previous studies have also shown that TNF-α blockers increase the risk of NTM infection (56–59). Winthrop et al. identified that the rates of NTM infection were significantly higher in rheumatoid arthritis patients than in age-matched patients without rheumatoid arthritis from 2000 to 2008 in northern California (56). Moreover, the rates of NTM infection were significantly higher in rheumatoid arthritis patients receiving anti-TNF-α therapy than in those not receiving the therapy (56). Two studies from South Korea revealed a high incidence of NTM disease in patients receiving anti-TNF-α therapy (60, 61).

Corticosteroids are broadly used to downregulate inflammation in chronic diseases, such as chronic obstructive pulmonary disease (COPD), asthma, and rheumatoid arthritis (99). Similar to TNF-α blockers, a high incidence of NTM diseases has been observed in patients receiving corticosteroid therapy. Andréjak et al. (2013) identified that the incidence of NTM diseases was higher in COPD patients currently receiving inhaled corticosteroids (ICs) than in COPD patients previously receiving ICs or patients not using ICs in northern Denmark (100). In addition, Dirac et al. revealed that several host traits, such as COPD, steroid use, and pneumonia hospitalization, seem to be associated with MAC lung disease susceptibility (39). Similarly, the risk of NTM diseases was greater in asthmatic patients receiving long-term, high-dose IC therapy (62). Taken together, the prolonged use of immunosuppressive drugs likely increases the susceptibility to NTM (**Table 1**).

#### Vitamin D deficiency

Vitamin D is an essential nutrient involved in the regulation of calcium homeostasis and metabolism (101). It induces the differentiation of premature monocytes into phagocytic macrophages (102). Vitamin D also activates macrophages, thus promoting pathogen clearance (103), and induces cathelicidin production *via* interactions with the vitamin D receptor (VDR), retinoic acid, and retinoid X receptor. Cathelicidin production promotes autophagy induction and autophagosome maturation. Accumulating evidence suggests that vitamin D is crucial for antimycobacterial host response (63, 104–106). It promotes the production of antimicrobial peptides *via* TLR and IFN-γ signaling (104, 105). This process induces autophagy and phagosome maturation, thereby inhibiting the intracellular growth of mycobacteria (107). Associations between VDR polymorphisms and NTM infections have been previously reported. Gelder et al. investigated three types of VDR polymorphisms in 56 patients with *M. malmoense* pulmonary disease and 101 healthy controls (108). The prevalence of *ApaI A, TaqI t*, and *At VDR* haplotypes was increased, while that of FokI f was decreased in the patients (108). Contrasting observations were reported by Tanaka et al. where the VDR genotype analyzed in 111 patients with MAC lung disease and 177 healthy controls (109) revealed no significant difference in the prevalence of FokI and *TaqI* VDR polymorphisms between the groups (109).

In addition to VDR polymorphisms, several studies have reported that vitamin D deficiency is associated with the onset of NTM disease (63–66). Jeon et al. demonstrated that patients with NTM lung disease had lower serum levels of 25-hydroxyvitamin D than those in healthy controls (66). Additionally, the prevalence of severe vitamin D deficiency, defined as serum 25-hydroxyvitamin D level <10 ng/ml, was significantly higher in patients with NTM lung disease (66). Kim et al. reported the differential expression of vitamin D metabolite and antimicrobial peptides (AMPs) in NTM patients (63). The serum levels of cathelicidin and human β defensin-2 were not significantly different between patients and controls (63). Interestingly, the serum concentration and gene expression levels of cathelicidin, human β defensin-2, and vitamin D-binding protein of NTM patients were not correlated with serum vitamin D levels (63). The production of cathelicidin is mediated by the cleavage of hCAP-18 with proteinase 3 in neutrophils (63). Although the higher gene expression of cathelicidin was detected in NTM patients, the serum level of AMPs may not have been elevated due to the impaired extracellular cleavage of the hCAP-18 protein in NTM patients (63). Furthermore, if most of the produced AMPs are present in macrophage phagosomes, the serum level of AMPs may not be elevated (63). Fujita et al. reported that patients with MAC lung disease showed low serum levels of human cationic antimicrobial protein 18/cathelicidin; however, the serum vitamin D levels were not significantly different between patients and controls (64). Kwon et al. demonstrated that bioavailable vitamin D levels were associated with the development of NTM after adjusting for age, sex, body mass index (BMI), and vitamin D-binding protein levels; however, no significant changes in bioavailability were observed during treatment (65). One possible explanation for the different results between studies is that the production of vitamin D is affected by various factors. Diverse factors, such as dietary intake, degree of sunlight exposure, and medications, influence the systemic level of vitamin D (101). Therefore, factors influencing vitamin D production, such as diet, sun exposure, vitamin D supplementation, and medications, should be investigated in MAC lung disease patients to develop potential HDT.

#### TLR2 polymorphism

TLRs are PRRs that mediate the recognition of foreign antigens by the innate immune system (110). TLR2 recognizes mycobacterial antigens such as glycolipids, lipoarabinomannans, and lipoproteins of Mtb in macrophages (111, 112). Previous studies have reported an association between TLR2 expression and susceptibility to NTM disease. Ryu et al. evaluated the transcriptional levels of TLR2, TNF-α, and IL-12 p40 in PBMCs isolated from patients with NTM lung disease (23). The transcriptional level of TLR2 in PBMCs was lower in patients with NTM lung disease. Moreover, IL-12 p40 and TNF-α mRNA and the protein levels were lower in patients than in healthy controls (23). The same group also investigated the occurrence of TLR2 polymorphisms in patients with NTM lung disease and showed that TLR2 Arg677Trp and Arg753Gln polymorphisms were not associated with host susceptibility to NTM lung disease (67). Yim et al. evaluated the association between a guanine–thymine (GT) repeat polymorphism in TLR2 and NTM lung disease (68). In total, the analysis of 193 NTM patients and 191 healthy controls showed that the prevalence of shorter GT repeats was significantly higher in the NTM group than that in the control group (68). Furthermore, genotypes with S alleles were more common in MAC lung disease patients but not in patients with *M. abscessus* lung disease (68). Taken together, TLR2 and its polymorphisms may contribute to the progression of MAC lung disease.

#### Low BMI

Adipokines, such as leptin and adiponectin, are signaling molecules secreted by adipose tissue and play an essential role in energy metabolism, inflammation, and obesity (113). Adiponectin is a peptide hormone that plays a central role in obesity-associated diseases, such as type 2 diabetes, stroke, myocardial infarction, and atherosclerosis (114). It also plays a central role in regulating innate immunity and inflammation (115). Extensive evidence suggests that adiponectin plays an anti-inflammatory role by modulating macrophage proliferation and polarization (115–118). It facilitates M2 macrophage polarization and the upregulation of anti-inflammatory molecules such as IL-10 and Arg-1 (115) (**Table 1**). Conversely, adiponectin suppresses M1 macrophage polarization and the expression of pro-inflammatory cytokines, including TNF-α, MCP-1, and IL-6 (115) (**Table 1**). Several reports have shown that adiponectin levels are associated with the onset and progression of NTM-associated lung disease. Serum adiponectin levels were significantly higher in patients with MAC lung disease than in healthy controls, and the adiponectin concentration in both the patient and control groups was inversely proportional to BMI (69). In addition, Kim et al. reported that patients with lower abdominal fat showed a faster progression of MAC lung disease (70). This observation agrees with previous studies showing that a thinner chest subcutaneous fat or a low BMI was involved in the radiologic severity of MAC lung disease (71, 119). Similarly, BMI at the time point before diagnosis was significantly lower in patients with MAC lung disease than in healthy controls (72). The BMI of patients with MAC lung disease was significantly lower than that of patients with disseminated NTM (72).

Leptin is an adipocyte-derived factor that counteracts ghrelin-mediated hunger (120). Leptin plays multiple roles in innate immunity, including phagocytosis and pro-inflammatory cytokine production in macrophages (121). In addition, leptin mediates DC maturation and the activation and chemotaxis of basophils, eosinophils, and neutrophils to sites of infection and inflammation (121). Low leptin levels induce high susceptibility to NTM infection (122) (**Table 1**). In addition, a low number of adipocytes reduce TNF-α and IL-6 levels and impair Th17 cell function, leading to increased susceptibility to mycobacterial infection (123, 124). Furthermore, leptin levels are associated with the upregulation of pro-inflammatory cytokines (125). Postmenopausal women with a low BMI are more susceptible to NTM infection due to decreased leptin levels (125). However, Tasaka et al. reported that the levels of TNF-α and IL-6 were higher in the serum of patients with MAC lung disease and that the levels of leptin in the serum were markedly lower than those in the control group (69). Furthermore, no significant correlation between BMI and leptin was observed (69). Taken together, leptin and adiponectin appear to play multiple roles inthe pathogenesis of mycobacterial infections. Further studies in this regard are essential to clarify the role of adipokines in modulating macrophage function in MAC lung disease.

#### Aging

Extensive evidence suggests that MAC infection rates increase with age (126–132). Aging degenerates the functional activity of the immune system, resulting in a high susceptibility to infection (73). Previous studies have revealed that aging deteriorates macrophage function in several ways.

First, aging modulates cytokine production by stimulating TLR and IFN-γ (**Table 1**). Renshaw et al. showed that splenic and peritoneal macrophages isolated from aged mice (18–24 months old) produced significantly lower amounts of TNF-α and IL-6 than those isolated from young mice (2 to 3 months old) in response to diverse TLR ligands, such as LPS, poly(I:C), zymosan A, *Staphylococcus aureus*, and flagellin from *Salmonella typhimurium* (74, 75). The expression of p38 MAPK and c-jun NH2-terminal kinase was lower in peritoneal macrophages from aged mice than those from young mice (75). Furthermore, aged mice showed lower LPS- and zymosan-induced TNF-α and IL-6 production in splenic macrophages compared with young mice (76). Additionally, the expression of nuclear factor (NF)-κB, but not IL-2, was significantly downregulated in splenic macrophages from aged mice compared with that in macrophages from young mice after LPS stimulation (76). These observations suggest that the macrophages of aged mice show repressed pro-inflammatory cytokine production and MAPK signaling pathways.

Second, aging modulates macrophage polarization (**Table 1**). Polarization of both M1 and M2 types was decreased in splenic macrophages (77). The expression of nitric oxide synthase (iNOS) was significantly lower in splenic macrophages from aged mice than in those from young mice after TNF-α and IFN-γ stimulation (77). Similarly, the expression of IL-1β and TNF-α was significantly lower in aged mice than in young controls after LPS stimulation (77). Moreover, the expression of several M2 markers, such as Arg1, FIZZ1, and Ym1, was significantly lower in aged mice than in young mice after IL-4 stimulation (77). In contrast, a shift towards M2 polarization was observed in aged mice in several studies. According to Kelly et al., ocular macrophages from aged mice showed M2-like phenotypes, including upregulation of IL-10 and downregulation of IL-12 and TNF-α (78). In addition, a high proportion of M2 macrophages was found in the spleen, lymph nodes, and bone marrow of aged mice (133).

Third, aging affects the phagocytic function of macrophages (**Table 1**). Reduced phagocytosis was observed in aged rats infected with *S. typhimurium* and *Trypanosoma musculi* (133, 134). Similarly, the peritoneal macrophages from aged BALB/c mice displayed impaired phagocytosis (135). Aprahamian et al. revealed that serum from aged mice induces a reduction in phagocytosis in peritoneal macrophages (136). Interestingly, aging attenuates the phagocytosis of peritoneal macrophages but not bone marrow-derived macrophages and suggests that the high susceptibility to NTM infection in the elderly population may be associated with aging-related dysfunction of tissue-resident alveolar macrophages (79). These observations suggest that microenvironmental factors in specific tissues determine the aging-associated dysfunction of macrophages (73). Therefore, understanding the microenvironment alteration with age is essential for improving the immune function of macrophages in the elderly to control MAC infection.

## HDTs modulate macrophage functions for MAC infection

The host susceptibility factors of MAC infection have allowed a deeper understanding of the host–pathogen interactions in MAC, confirming the importance of host immunological function in MAC infection. Pro-inflammatory response aids the clearance of MAC *via* the IL-12/IFN-γ axis and associated signal transduction pathways. However, excessive inflammation causes lung injury, leading to a poor prognosis. Therefore, modulation of the balance between pro- and anti-inflammatory responses is critical for controlling MAC infection without detrimental effects on the host. In this respect, enhancement of the antimycobacterial activity of macrophages that are manipulated or suppressed by MAC infection and modulation of excessive inflammation are promising strategies for the treatment of MAC infection. Based on the important signaling pathways in macrophages that contribute to MAC infection summarized previously, in this chapter, we propose HDTs with the potential to control MAC infection by improving antimycobacterial activity and modulating detrimental inflammation. HDTs applied to MAC infection are still rare; therefore, many cases have been described based on the contents applied to Mtb infection. The potential HDT strategies applied or that are applicable to MAC infections are summarized in **Table 2**.

**Table 2:** Proposed list of potential host-directed therapeutics to modulate macrophage function against *Mycobacterium avium* complex infection.

Host-directed therapy agents	Host target	Effect to macrophages	Subject	Therapeutic outcome	References
Metformin	Autophagy Reactive oxygen species (ROS) production Phagosome maturation	Promotion of mitochondrial ROS production, autophagy, mTORC1 inhibition, phagosome–lysosome fusion, and apoptosis	Human monocyte-derived macrophages (MDMs) THP-1 cells	Reduction of *M. tuberculosis* (Mtb) survival	(137)
			Mouse (C57BL/6)	Reduction of lung tissue pathology	
			Human peripheral blood mononuclear cells (PBMCs)	Inhibition of Mtb-induced type I IFN response and inflammation	(138)
			THP-1 cells	Enhancing phagocytosis Inhibition of Mtb-induced type I IFN response and inflammation	(139)
			THP-1 cells	Reduction of Mtb survival	(140)
			Guinea pig (Dunkin–Hartley)	Reduction of lung lesion and Mtb burden	(141)
Statin	Autophagy Phagosome maturation	Induction of autophagy, phagosome maturation, phagosome–lysosome fusion, and apoptosis	PBMCs and MDMs from patients with familial hypercholesterolemia	Reduction of Mtb burden	(142)
			Human PBMCs	Reduction of Mtb burden	(143)
			THP-1 cells	Improves the bactericidal effect of first-line antibiotics	(144)
			Mouse (BALB/c)	Shortening of treatment duration	
			THP-1 cells	Improves the bactericidal effect of first-line antibiotics	(145)
			Mouse (C3HeB/FeJ and BALB/c)	Promotion of bacterial clearance Reduction of lung pathological lesion	
			Human bone-marrow-derived macrophages (BMDMs)	Growth arrest of *M. avium*	(146)
Resveratrol	Autophagy Phagosome maturation	Suppression of Mtb-induced apoptosis Induction of autophagy, phagosome–lysosome fusion	Mouse (Sirt1^+/−^ and TLR2^−/−^)	Reduction of lung Mtb loads and pathological lesions	(147)
			Murine peritoneal macrophages	Reduction of Mtb survival	(148)
			THP-1 cells and human MDMs	Reduction of Mtb survival	(149)
			Mouse (C57BL/6)	Restriction of Mtb growth Reduction of Mtb-induced tissue pathology and inflammation		
Valproic acid	HDAC1	Pro-inflammatory cytokines and nitric oxide (NO) production	Macrophages (J774)	Reduction of Mtb survival		(150)
							THP-1 cells	Reduction of Mtb survival	(151)
			Curcumin	NRF2 signaling		Induction of phagosome–lysosome fusion	THP-1 cells	Reduction of Mtb survival	(152)
			Sulforaphane	NRF2 signaling		Induction of phagosome–lysosome	Mouse alveolar macrophages	Reduction of *M. avium* survival	(153)
			NAC and glutathione	ROS production NF-κB signaling		Inhibition of excessive ROS production Decreased production of pro-inflammatory cytokines	THP-1 cells and human MDMs	Reduction of *Mtb*, *M. avium*, and *M. bovis* Bacillus Calmette–Guerin (BCG) survival	(154)
							Mouse (C57BL/6)	Reduction of lung Mtb burden	
							*In vitro* granuloma	Reduction of Mtb survival	(155)
							*In vitro* granuloma	Reduction of Mtb survival	(156)
							*In vitro* granuloma	Reduction of *M. bovis* BCG survival	(157)
							THP-1 cells	Reduction of Mtb survival with restored glutathione level	(158)
							THP-1 cells and human PBMCs	Promotion of intracellular killing of both BCG and virulent Mtb strain	(159)
							RAW264.7 cells	Reduction of intracellular survival of *M. avium*	(160)
							Mouse (BALB/c)	Reduction of lung bacteria burden		
			Arginine and citrulline	NO production		Increased intracellular bacterial killing	Clinical trial	Induction of higher sputum conversion rate, increased body weight, and reduction of cough in HIV-negative TB patients		(161)
							Clinical trial	Reduction of constitutional symptoms, increased body mass index (BMI), and decreased C-reactive protein level in pulmonary TB patients		(162)
							Clinical trial	No significant change of reduction of constitutional symptoms, increased BMI, and decreased C-reactive protein level in pulmonary TB patients		(163)
			Ursolic acid	NO and ROS production		Increased production of pro-inflammatory cytokines M1 macrophage polarization	Mouse (BALB/c)	Reduction of lung Mtb burden and pneumonic area		(164)
							J774A.1 cells	Reduction of Mtb survival		
							THP-1 cells	Reduction of Mtb survival		(165)
							Murine BMDMs	Reduction of *M. avium* survival		(166)
			TNF-α blockers	TNF-α signaling		Decreased production of pro-inflammatory cytokines	Mouse (C3HeB/FeJ)	Reduction of lung Mtb burden and pathological lesion		(167)
											Case report	Reduction of pathological lesion and clinical symptoms	(168)
							Corticosteroids	Immunomodulatory transcription factors		Promotion of anti-inflammatory phenotype Inhibition of necrotic cell death	Clinical trial	Reduction of excessive inflammation	(169)
														Clinical trial	Improvement of survival rate	(170–172)
											NSAIDs	Immunomodulatory transcription factors	Suppression of prostaglandin E2	Mouse (C3HeB/FeJ)	Ibuprofen significantly increased the survival rate Reduction of lung Mtb burden and pathological lesion	(173)
														Mouse (BALB/c)	Ibuprofen enhanced intracellular killing	(174)
														Clinical trial	No beneficial effect	(175)
														Murine peritoneal macrophages	No significant change of intracellular survival	(176)
														Mouse (BALB/c)	No significant change of lung bacterial burden	

### HDT agents enhance the antimycobacterial activity of macrophages

#### Metformin modulates ROS production and phagosome maturation

**Figure 3:**
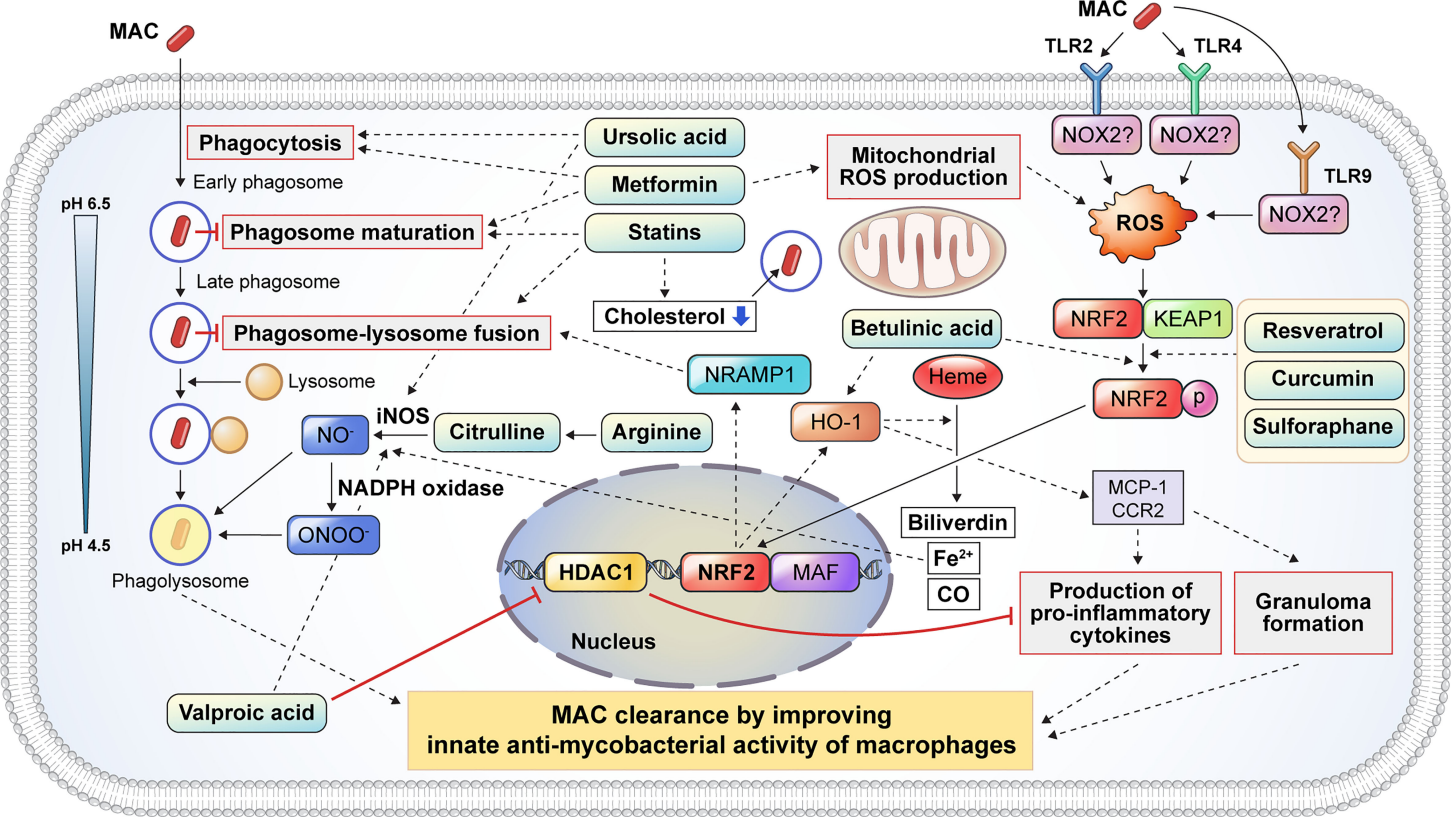
Proposed host-directed therapeutic agents enhance the host innate antimycobacterial activities against MAC infection. Several inflammatory signaling pathways are activated to eliminate intracellular mycobacteria. Host-directed therapeutic drugs may improve the clinical outcome of MAC infection by modulating the antimycobacterial response of macrophages, including phagocytosis, phagosome maturation, reactive oxygen and nitrogen species production, pro-inflammatory cytokine production, and granuloma formation. Metformin and UA promote the phagocytosis of extracellular mycobacteria. After phagocytosis, mycobacteria-containing phagosomes mature through a series of fusion and fission events with endosomes before fusing with lysosomes. Several drugs, including statins and metformin, promote phagosome maturation. Additionally, statins induce phagosome–lysosome fusion and cholesterol depletion to promote the intracellular killing of MAC. Citrulline and arginine promote nitric oxide (NO) production following the conversion of arginine to citrulline in macrophages *via* iNOS. Furthermore, UA and VA promoted the production of NO by upregulating iNOS. VA inhibits the expression of HDAC1, which suppresses the production of pro-inflammatory cytokines. Mycobacterial infection induces ROS production through the activation of TLR2 and TLR4 signaling which initiates the dissociation of KEAP1 from NRF2. Several drugs, such as resveratrol, curcumin, and betulinic acid, promote the dissociation of NRF2 from KEAP1, leading to the nuclear translocation of NRF2 which activates NRAMP1 and HO-1. The activation of NRAMP1 promotes phagosome–lysosome fusion, leading to the intracellular killing of mycobacteria in macrophages. In addition, HO-1 activation promotes granuloma formation, which restricts mycobacterial infection. HO-1 catalyzes heme into biliverdin, Fe^2+^, and CO. Fe^2+^ promotes NO production from arginine *via* iNOS. HO-1 also promotes the production of pro-inflammatory cytokines and granuloma formation through MCP1 and CCR2 signaling. In summary, the induction of phagocytosis, phagosome maturation, ROS/NO production, NF-κβ signaling, NRF2–KEAP1, and HO-1 pathways by numerous drugs facilitates the restriction and clearance of MAC and subsequently improves the clinical outcomes. MAC, *Mycobacterium avium* complex; UA, ursolic acid; iNOS, inducible nitric oxide synthase; VA, valproic acid; HDAC1, histone deacetylase 1; ROS, reactive oxygen species; TLR, Toll-like receptor; KEAP1, Kelch-like ECH-associated protein 1; NRF2, nuclear factor-erythroid factor 2-related factor 2; NRAMP1, natural resistance-associated macrophage protein 1; HO-1, heme oxygenase-1; MCP1, monocyte chemoattractant protein 1; CCR2, C-C chemokine receptor type 2; NF, nuclear factor.

Metformin (MET) is a Food and Drug Administration-approved medication for the treatment of type 2 diabetes mellitus (177). MET has multiple mechanisms that improve the clinical outcomes of TB, especially in patients with diabetes. MET induces mitochondrial ROS production, phagocytosis, and phagosome maturation, facilitating pathogen clearance in Mtb-infected macrophages (**Figure 3**). Singhal et al. reported that MET induced a substantial reduction in Mtb survival by enhancing mitochondrial ROS production and phagosome maturation in macrophages (137). In addition, MET promotes the resolution of pathological lesions and reduces the bacterial burden in Mtb-infected mice (137) (**Table 2**). MET also modulates cellular metabolism and cytokine production in Mtb lysate-stimulated PBMCs (138). The administration of MET induces a substantial reduction in oxidative phosphorylation, mammalian target of rapamycin signaling, and type I interferon response pathways in PBMCs after stimulation with Mtb lysate without reducing cellular viability (138). MET also improves antimycobacterial immunity by enhancing ROS production and phagocytosis in macrophages (138) (**Table 2**). Similarly, Cervantes et al. showed that MET enhanced the phagocytic ability of macrophages during Mtb infection and downregulated type I IFN pathways that promote TB pathogenesis (139). Frenkel et al. reported that MET induced a reduction in inflammatory cytokines, promoting the resolution of Mtb-induced lung pathology that is detrimental to the host in a guinea pig model (141) (**Table 2**). Furthermore, MET has been shown to reduce the bacterial loads in macrophages and lung epithelial cells by producing host defense peptides, such as β-defensin-2, β-defensin-3, and β-defensin-4 (140). These findings suggest that MET provides host protection by reducing Mtb-induced pathological lesions *via* the modulation of inflammation and metabolism (**Table 2**). MET also enhances the anti-TB immune response of macrophages, including ROS production, phagosome maturation, and phagocytosis. Therefore, MET is a promising HDT drug for mycobacterial infections with respect to host protection and pathogen clearance. However, the potential role of MET in MAC infection remains unknown, and further research is needed to investigate its suitability for improving clinical outcomes in MAC infection.

#### Statins modulate phagosome maturation and lipid accumulation

Statins are cholesterol-lowering drugs that inhibit the 3-hydroxy-3-methylglutaryl coenzyme A reductase of host cholesterol synthesis and subsequently prevent heart attacks and stroke (178). Previous studies have revealed that statins promote bacterial clearance in Mtb infection by enhancing phagosome maturation and intracellular cholesterol depletion in macrophages (**Figure 3**). Statin therapy reduces the bacterial burden in PBMCs and monocyte-derived macrophages from familial hypercholesterolemia (142). Furthermore, statin-induced depletion of cholesterol inhibits the arrest of phagosome maturation caused by Mtb infection (142). Moreover, statin therapy provides host protection with a substantial reduction in lung bacterial load and pathological lesions in a murine model (142) (**Table 2**). Similarly, simvastatin itself had no direct killing activity against Mtb, promoting bacterial clearance in Mtb-infected PBMCs *via* the formation of autophagosomes (143). In addition, simvastatin increased the expression of co-stimulatory molecules, production of IL-1β and IL-12p70, apoptosis, and autophagy in Mtb-infected monocytes (143). Simvastatin potentiates the activity of first-line anti-TB drugs, such as rifampin, isoniazid, and pyrazinamide, in Mtb-infected macrophages and mice (144). In addition, statin therapy reduces the duration to achieve lung culture negativity in Mtb-infected BALB/c mice (144) (**Table 2**). Statins, such as simvastatin, fluvastatin, and pravastatin, potentiate the activity of isoniazid, rifampin, and pyrazinamide in Mtb-infected THP-1 cells (145) (**Table 2**). Pravastatin promotes the phagosomal acidification and proteolytic activity of Mtb-infected macrophages, thereby enhancing their intracellular killing ability (145). These results suggest that statins improve the resistance to Mtb infection in macrophages by promoting autophagy and phagosome maturation. Furthermore, statins decrease the lipid droplet formation in macrophages, which serves as a nutrient source for the intracellular survival of Mtb (179). MAC infection also induces lipid accumulation in macrophages, similar to Mtb infection, which leads to the growth arrest of intracellular *M. avium* (146). Although the role of lipid droplets in foamy macrophages in MAC pathogenesis is less well understood than that in TB, statins may increase innate resistance to MAC in macrophages. However, further investigation is required to identify the effects of statins on MAC infections.

#### Arginine and citrulline enhance NO production

Arginine is a dibasic α-amino acid that modulates diverse biological processes, such as vasodilation, neurotransmission, wound healing, endocrine secretion, and immunity (180–182). Arginine-mediated nitric oxide (NO) production kills a variety of pathogens and thus plays a key role in innate immune response (181). Therefore, arginine deficiency may increase host susceptibility to infection. Arginine synthesis is critical for the host immune response during mycobacterial infection (183–185). During infection, extracellular arginine is imported to generate NO in macrophages, thereby increasing the level of citrulline, and is not recycled to produce arginine (185). Depletion of arginine induces the import of extracellular citrulline and consequently produces arginine by argininosuccinate synthase and argininosuccinate lyase for optimal NO production in macrophages (185). Qualls et al. showed that impaired arginine synthesis increased the Mtb burden in macrophages (185). Rapovy et al. demonstrated that citrulline was a more favored source of NO production than arginine in arginase-expressing murine macrophages for the induction of an antimycobacterial host response (183). iNOS and arginase are expressed in macrophages isolated from human TB granulomas (186). Arginine supplementation improves the clinical outcome of patients with TB by enhancing the NO-dependent antimycobacterial activity. Schön et al. reported that arginine induced a higher sputum conversion rate, increased the body weight, and reduced the cough in HIV-negative TB patients (**Table 2**), but not in HIV-positive TB patients (161). Farazi et al. demonstrated that arginine supplementation promoted the reduction of constitutional symptoms, increased the BMI, and decreased the C-reactive protein levels in pulmonary TB patients (162) (**Table 2**). In contrast, Ralph et al. reported that arginine supplementation did not improve clinical outcomes such as sputum culture conversion rate, weight change, and clinical symptoms (163) (**Table 2**). Interestingly, contrasting results have been reported in *M. avium*-infected murine and human macrophages to identify the role of NO in MAC pathogenesis (187–189). Doi et al. reported that L-arginine-induced NO production significantly suppressed the bacterial growth in IFN-γ-treated rat alveolar macrophages (188). However, Bermudez et al. demonstrated that the depletion or inhibition of arginine did not influence *M. avium* growth in murine peritoneal macrophages or human monocyte-derived macrophages (187). Similarly, NO production did not inhibit the growth of *M. intracellulare* in rat macrophages (189). To date, the relationship between NO production and MAC infection has not yet been fully elucidated. Therefore, further studies are needed to identify the role of arginine metabolism in MAC infection.

#### Ursolic acid promotes phagocytosis and ROS/NO production

Ursolic acid (UA) is a natural triterpene that is abundant in vegetables, medicinal herbs, and fruits (190). UA has been reported to modulate diverse physiological processes such as inflammation, neoplastic growth, and antimicrobial immune response (164, 191). It has been shown to promote phagocytosis, autophagy induction, and ROS production in macrophages (**Figure 3**). Previous studies have revealed that UA promotes the intracellular killing effect of macrophages in mycobacterial infection (164–166, 192, 193). UA treatment significantly reduced the lung Mtb burden and pneumonic lung area (164) (**Table 2**). In addition, the expression of IFN-γ and TNF-α did not differ between UA-treated and untreated mice (164). López-García et al. demonstrated that UA treatment inhibited intracellular Mtb growth in macrophages, with increased production of NO and ROS (192) as well as increased expression levels of TNF-α, CD36, and TGR5 and decreased expression levels of TGF-β (192). These observations suggest that UA has immunomodulatory activities that activate macrophages. Specifically, treatment with UA converted the macrophage polarization status from an M2-like to an M1-like phenotype. Similarly, Podder et al. showed that UA promoted the intracellular killing of Mtb in THP-1 macrophages by enhancing ROS and NO production (165) (**Table 2**). The induction of ROS and NO production was also observed in Mtb-infected human alveolar epithelial cells after treatment with UA (193). UA treatment also promoted the intracellular killing of *M. avium* in macrophages (166) (**Table 2**). Furthermore, UA inhibited the expression of TNF-α and IL-6 and promoted the expression of IL-1β and nitrite. It inhibited the phosphorylation of ERK1/2 and promoted the phosphorylation of the C-Jun N-terminal kinase signaling pathway (166). These observations suggest that UA may be a promising HDT target for TB and MAC infections. However, the clinical suitability of UA as an HDT target in MAC infection should be investigated in future studies.

#### Nuclear factor-erythroid factor 2-related factor 2–Kelch-like ECH-associated protein-1 signaling modulating drugs

Dysregulation of the balance between oxidants and antioxidants within cells causes oxidative stress, with elevated levels of free radicals and other reactive species (194). Free radicals and reactive species damage intracellular proteins, lipids, and DNA (195). Nuclear factor-erythroid factor 2-related factor 2 (NRF2) is a widely conserved factor which mediates the defense mechanism against oxidative stress through transcriptional regulation (195). Under non-oxidative stress conditions, NRF2 is localized in the cytoplasm along with Kelch-like ECH-associated protein-1 (KEAP1) (196) (**Figure 3**). Under oxidative stress, NRF2 translocates into the nucleus and forms a heterodimer with small musculoaponeurotic fibrosarcoma proteins and binds to the antioxidant response element consensus sequence for transcriptional regulation (196) (**Figure 3**). To date, only a few studies have investigated the role of NRF2–KEAP1 signaling in NTM infection. *M. abscessus* infection induces ROS production and necrosis despite the activation of NRF2–KEAP1 signaling in THP-1 macrophages (197). In addition, activation of NRF2 signaling by sulforaphane significantly promotes the reduction of intracellular survival of *M. abscessus* through apoptosis induction (197). Recently, Nakajima et al. showed that the survival rate of Nrf2-deficient mice was significantly lower than that of wild-type mice with MAC infection (153). Although the degree of inflammation was not altered between Nrf2^−/−^ and wild-type mice, the size of the granulomas was significantly lower in Nrf2^−/−^ mice (153). Interestingly, Th1 immunity and oxidative stress levels did not differ between Nrf2^−/−^ and wild-type mice (153). Transcriptional analysis revealed that NRF2 regulates the expression of natural resistance-associated macrophage protein 1 (NRAMP1) and HO-1 in MAC-infected alveolar macrophages and mice (153). NRAMP1 expression induced by NRF2 translocation promotes phagosome–lysosome fusion and subsequently induces the killing of intracellular mycobacteria (**Figure 3**). The upregulation of HO-1 enhances the degradation of heme into carbon monoxide, biliverdin, and free iron (198). Free iron induces NO production *via* iNOS, thereby promoting pathogen clearance (**Figure 3**). In addition, activation of NRF2 by sulforaphane promotes the intracellular killing of MAC in alveolar macrophages by activating the phagosome–lysosome fusion (153). Taken together, these data suggest that the activation of NRF2 signaling provides a host-beneficial effect in MAC infection.

Betulinic acid (BA) is a natural pentacyclic triterpenoid derived from various plants with antiretroviral, antitumor, and anti-inflammatory properties (199, 200). BA shows antimicrobial activity against *Mycobacterium* species (201–203). In addition to its direct antimycobacterial activity, BA promotes bacterial clearance during mycobacterial infection through the activation of the NRF2–KEAP1 and HO-1 signaling pathways (**Figure 3**). Treatment with BA induces the nuclear translocation of NRF2, and its activation enhances the expression of NRAMP1, leading to increased phagosome–lysosome fusion (**Figure 3**). BA also promotes pro-inflammatory cytokine production and granuloma formation through the activation of HO-1 signaling (**Figure 3**).

Curcumin is a natural polyphenol derived from turmeric that has a potential role in diverse pathophysiological processes (204). It exhibits a variety of antifungal, antiviral, antioxidant, and anti-inflammatory responses (205). In addition, the antimycobacterial properties of curcumin have also been reported (152, 206). It promotes bacterial clearance through macrophages *via* the modulation of host immune responses (**Figure 3**). Pretreatment with curcumin induces a reduction in the intracellular Mtb burden in THP-1 cells and primary human alveolar macrophages (152). Furthermore, curcumin activates the nuclear translocation of NRF2, which leads to enhanced phagosome–lysosome fusion (**Figure 3**). The antimycobacterial activity of curcumin through the modulation of host innate resistance remains unclear. The effect of curcumin on MAC infection has not yet been elucidated. Taken together, BA and curcumin may contribute to bacterial clearance in MAC infection through the modulation of NRF2 and HO-1 signaling in macrophages, and their protective role against MAC infection needs to be identified.

#### Valproic acid

Valproic acid (VPA) is widely used for the treatment of seizures and bipolar disorder (207). In addition, VPA shows antimycobacterial activity in macrophages (150, 207). Treatment with VPA induces a higher production of NO and a significant reduction in intracellular Mtb burden in macrophages (150). Similarly, Rao et al. reported that VPA significantly reduced intracellular Mtb levels in macrophages (151). In addition, the combination of VPA, isoniazid, and rifampicin showed a synergistic effect compared with monotherapy (151). The potential mechanisms by which VPA manifests its antimycobacterial activity include NO production and histone deacetylase 1 (HDAC-1) inhibition (**Figure 3**). HDAC-1 inhibits NF-κB-mediated gene expression and subsequently suppresses pro-inflammatory responses. The inhibition of HDAC-1 by VPA induces the production of pro-inflammatory cytokines, which leads to bacterial clearance (**Figure 3**).

### HDT agents suppress excessive inflammation

#### Resveratrol

Resveratrol is a natural polyphenol that is abundant in grapes, berries, and other plants and has been shown to have anti-inflammatory, antioxidative, and antiproliferative effects (208, 209). Resveratrol differentially modulated immune function depending on its concentration (210). Low (high) concentrations of resveratrol activate (suppress) the immune response of T cells, B cells, and macrophages (210, 211). Resveratrol inhibits the activation of the TAK1, MAPK, and NF-κB pathways, thereby suppressing the production of pro-inflammatory cytokines such as IL-6 and TNF-α in Mtb-infected macrophages (147). Additionally, treatment with resveratrol significantly reduces lung bacterial loads and pathological lesions in Mtb-infected mice (147) (**Table 2**). Resveratrol treatment also inhibits the intracellular growth of Mtb *via* the activation of host sirtuin 1 in mouse peritoneal macrophages (148) (**Table 2**). Sirtuin 1 activation promotes the PI3K-induced phosphorylation of Akt and GSK3β, thereby suppressing Mtb-induced apoptosis (148). Interestingly, pretreatment with a GSK3β phosphorylation inhibitor promotes intracellular Mtb replication (148). Furthermore, sirtuin 1 deactivates RelA, which is the p65 subunit of NF-κB (149). NF-κB modulates various immune responses, including dendritic cell maturation, classical activation of macrophages, differentiation of Th1 cells, and production of pro-inflammatory cytokines (212). Cheng et al. demonstrated that Mtb infection induced a reduction of sirtuin 1 expression in macrophages and mouse models with NF-κB overexpression (149). Overexpression of NF-κB induces the inhibition of apoptosis and autophagy in human macrophages, thereby promoting the intracellular growth of Mtb (213). Activation of sirtuin 1 reduces the intracellular replication of drug-susceptible and drug-resistant Mtb strains in macrophages *via* the induction of phagosome–lysosome fusion and autophagy (149) (**Table 2**). Collectively, previous studies have suggested that resveratrol-induced sirtuin 1 activation promotes host protection in TB pathogenesis by inhibiting apoptosis, promoting phagosome–lysosome fusion, and autophagy induction. In addition to sirtuin 1 activation, resveratrol promotes the nuclear translocation of NRF2 from the cytoplasm (**Figure 3**). Activation of NRF2-induced bacterial clearance in *M. avium*-infected macrophages occurs through the expression of NRAMP-1 and HO-1 (153). However, the potential effect of resveratrol on MAC infections needs further investigation with emphasis on sirtuin 1 and NRF2 signaling.

#### N-acetyl-cysteine and glutathione

N-acetyl-cysteine (NAC) is a cysteine derivative commonly used as a mucolytic medication to induce expectoration (214). In addition to its mucolytic activity, NAC has been used as an antioxidant agent for the adjunctive therapy of infectious diseases and cancer (215–218). NAC induces an anti-inflammatory response *via* the inhibition of NF-κB, thereby reducing the production of pro-inflammatory cytokines, such as IL-6 and TNF-α, in macrophages (219, 220). Excessive oxidative stress induced by mycobacterial infection induces direct cell injury and pro-inflammatory immune response, leading to lung damage. Multiple studies have shown that NAC provides beneficial effects to the host against mycobacterial infection. Amaral et al. showed that oxidative stress and cell death induced by mycobacterial infection in Mtb-infected human macrophages were reduced by NAC treatment (154). NAC inhibits the metabolic activity and intracellular growth of Mtb, *M. avium*, and *M. bovis* BCG in a dose-dependent manner (154) (**Table 2**). Interestingly, NAC treatment significantly reduced the lung bacterial burden of Mtb-infected mice in both wild-type and gp91^Phox−/−^ mouse macrophages, suggesting that the antimycobacterial effect of NAC is not entirely dependent on NAPDH-induced ROS production within macrophages (154). Similarly, NAC treatment significantly reduced the intracellular survival of Mtb in *in vitro* granulomas, with enhanced production of IFN-γ (155) (**Table 2**). Furthermore, NAC promotes the restriction of Mtb within the acidified compartments of granulomas, thereby inhibiting the intracellular replication of Mtb (155). Glutathione (GSH), which is derived from NAC, protects cells from oxidative damage, thus maintaining redox homeostasis (221). GSH protects the host by inducing antioxidant activity, antigen degradation, and pro-inflammatory cytokine production. Mtb infection is also known to significantly reduce the intracellular level of GSH in macrophages (158). Low intracellular levels of GSH cause impaired secretion of IL-12 in macrophages, leading to diminished production of IFN-γ in NK cells (222). In turn, the decreased production of IFN-γ impairs the intracellular bactericidal activity of macrophages (223). Cao et al. showed that NAC treatment restored intracellular GSH levels in Mtb-infected macrophages and that NAC-induced restoration of GSH was associated with decreased intracellular survival of Mtb within macrophages (158). It has been recently reported by To et al. that GSH supplementation promotes the intracellular killing of mycobacteria in *in vitro* granulomas derived from patients with type 2 diabetes (156) (**Table 2**). This is mediated by the upregulation of Th1-type cytokines such as IFN-γ, TNF-α, and IL-2 while reducing the expression of IL-6 and IL-10 (222). A combination of everolimus and GSH significantly inhibits the intracellular survival of *M. bovis* BCG in *in vitro* granulomas derived from patients with type 2 diabetes (157) (**Table 2**). In addition, GSH increases the levels of Th1-type cytokines such as IFN-γ, TNF-α, and IL-2 as previously reported in Mtb infection (157). Liposomal GSH treatment promotes the intracellular killing of both BCG and virulent Mtb strains in THP-1 macrophages (159) (**Table 2**). GSH also reduces excessive pro-inflammatory cytokine production in *in vitro* granulomas derived from patients with type 2 diabetes and healthy controls (159). Taken together, accumulating evidence suggests that NAC-derived GSH promotes bacterial clearance by enhancing the microbicidal activity of macrophages and protects the host by reducing the overexpressed pro-inflammatory immune response. To date, only one group has investigated the effect of NAC treatment on MAC infection. Shiozawa et al. reported that NAC treatment induced a significant reduction in the intracellular survival of *M. avium* in a macrophage and mouse model, with increased expression of β-defensin-3 (160) (**Table 2**). However, NAC did not show a direct killing effect on *M. avium* culture (160). The NAC-induced clearance of mycobacteria disappeared in IL-17A-knockout mice, suggesting that IL-17 signaling may be associated with the modulation of NAC-induced β-defensin-3 production (160). Further studies are required to evaluate the potential role of NAC in host-directed therapies for MAC infections.

#### TNF-α blockers

Although the production of pro-inflammatory cytokines is critical for host immune responses, excessive inflammation has a detrimental effect on the host (224). Therefore, proper regulation of inflammation is required to restrict mycobacterial infection and prevent destructive pathological effects. TNF-α promotes the intracellular killing of macrophages during mycobacterial infection by inducing the apoptosis of infected cells (225, 226). However, overexpression of TNF-α induces excessive inflammation, thereby promoting tissue destruction *via* mitochondrial ROS production and necrosis in macrophages (227, 228). A variety of TNF-α blockers have been used for autoimmune diseases, such as rheumatoid arthritis, inflammatory bowel disease, and multiple sclerosis (229). TNF-α blockers affect the binding of soluble TNF to the TNF-α receptor and inhibit signal transduction pathways such as NF-κB. Treatment with TNF-α inhibitor, etanercept, significantly reduced the lung Mtb burden and TB-induced pathological lesions in mice (167) (**Table 2**). Wallis et al. (2009) reported that adalimumab treatment improved the clinical outcomes of patients with life-threatening TB (168) (**Table 2**). In addition, discontinuation of infliximab treatment in patients with TB induced poor clinical outcomes with mediastinal lymphadenopathy and multiple intrapulmonary miliary lesions (173, 230). However, the suitability of TNF-α blockers as an HDT for mycobacterial infections remains controversial. Multiple studies have reported that the risk of mycobacterial infection is elevated during anti-TNF therapy (57, 61, 231). The understanding of the complex roles of TNF-α in MAC infections is still limited, suggesting a need for elucidating the role of TNF-α signaling as an HDT during MAC infection.

#### Corticosteroids and non-steroidal anti-inflammatory drugs

The innate immune response is critical for the control of TB. However, chronic inflammation caused by TB is detrimental to the host, causing tissue-destructive pathological lesions. Therefore, resolution of chronic inflammation is needed to improve the clinical outcomes of TB. Corticosteroids and non-steroidal anti-inflammatory drugs (NSAIDs) are potential candidates for HDT strategies to reduce tissue-destructive inflammation in TB. Corticosteroids have been used in patients with TB to reduce mortality by modulating pathological inflammation (232). According to Grab et al., dexamethasone inhibits necrosis of Mtb-infected macrophages, thereby enhancing the survival of infected cells (233). It inhibits necrotic cell death *via* the inhibition of p38 MAPK phosphorylation, thereby providing protection (233). Treatment with dexamethasone was found to significantly reduce TLR4 and MyD88 expression in monocytes derived from patients with tuberculous meningitis (TBM) (169). The downregulation of TLR4 and MyD88 may reduce excessive inflammation in patients with TBM, thus preventing brain edema, fibrosis, and cranial nerve damage (169) (**Table 2**). Similarly, dexamethasone treatment significantly improved the survival of TBM patients (170–172) (**Table 2**).

NSAIDs reduce uncontrolled inflammation by suppressing the recruitment of neutrophils, thereby preventing the exacerbation of TB (234). They block prostaglandin synthesis by inhibiting cyclooxygenase and lipoxygenase, subsequently affecting inflammatory signaling and suppressing the production of prostaglandin E2, which inhibits phagocytosis and the intracellular killing of macrophages (235). As shown by Vilaplana et al., ibuprofen treatment significantly increases the survival rate of Mtb-infected mice (173) (**Table 2**). In addition, it significantly reduces lung bacterial burden and pathological lesions (173). Similarly, Byrne et al. reported that treatment with ibuprofen enhanced the intracellular killing effect of isoniazid in Mtb-infected mice (236) (**Table 2**); however, aspirin antagonizes the effects of isoniazid (236). Both ibuprofen and aspirin enhance the antimycobacterial activity of pyrazinamide (174). Schoeman et al. evaluated the effect of aspirin on mortality and morbidity in children with TBM (175) (**Table 2**); treatment with aspirin did not have a significant beneficial effect on the subjects, and severe neurological involvement in the aspirin-treated group may have influenced the clinical outcome. Thus, further investigation is required to clarify this aspect. Treatment with diclofenac modulated the cytokine expression in MAC-infected macrophages but did not affect the intracellular survival of MAC (176) (**Table 2**).

Although corticosteroids and NSAIDs may prevent the detrimental effects of excessive inflammation, they can also inhibit bacterial clearance. Therefore, the use of corticosteroids and NSAIDs as HDTs for mycobacterial infections remains controversial due to their immunosuppressive effects. The potential role of immunosuppressive drugs as HDT for MAC infections has not yet been identified. Further investigation is needed to elucidate the effects of corticosteroids and NSAIDs on MAC infection to modulate the immunologic balance with HDT.

## Other promising HDT pathways that require further investigation

### HIF-1α pathway

Hypoxia-inducible factor-1 alpha (HIF-1α) is a transcriptional regulator that responds to oxygen tension and plays pivotal roles in a wide range of cellular and physiological processes such as energy metabolism, erythropoiesis, angiogenesis, cell growth, and differentiation (237). Previous studies have shown that HIF-1α plays a critical role in the regulation of immune cell metabolism and activity (238–240). Bacterial infection and depletion of oxygen tension induces NF-κB expression in macrophages, subsequently promoting HIF-1α expression (238, 240). Stabilization of HIF-1α enhances the bacterial killing activity of macrophages by promoting NO and TNF-α production (239).

Recent works have suggested that HIF-1α regulates the host innate immune response in mycobacterial infections (241–246). Cardoso et al. demonstrated that HIF-1α inactivation induces detrimental outcomes such as increased bacterial burden and the emergence of granuloma necrosis in *M. avium* infection (242). Braverman et al. also proved that HIF-1α activation is a pivotal regulator of the IFN-γ-dependent host response that modulates metabolism to eliminate Mtb in *in vitro* and *in vivo* settings (241). HIF-1α induces a metabolic shift to aerobic glycolysis in IFN-γ-activated macrophages, and increased glycolytic flux promotes the intracellular killing of mycobacteria by the production of IL-1β, ROS, and itaconate (247, 248). Marin Franco and colleagues revealed that the pleural effusion of TB patients induces detrimental effects to the control of TB infection, including reduced glycolytic flux, increased oxidative phosphorylation level, and bacterial burden (244). Moreover, the same group showed that HIF-1α stabilization by dimethyloxalylglycine (DMOG) modulates the macrophage phenotype by restoring M1 metabolism (244). Treatment with DMOG induced beneficial effects, with lowered bacterial burden due to the metabolic reprogramming of macrophages *via* HIF-1α pathway activation *in vitro* and *in vivo* (244). Similarly, Phelan et al. demonstrated that the stabilization of HIF-1α by the iron chelator desferrioxamine (DFX) improves antimycobacterial immune function by modulating immune-metabolism during the early stage of Mtb infection (245). DFX inhibits the degradation of HIF-1α by chelating iron in the cofactor of prolyl hydroxylase domain proteins (249). Stabilization of HIF-1α by DFX promotes the innate immune response by boosting glycolysis and the reduction of intracellular Mtb survival (245). Similarly, Zenk et al. revealed that HIF-1α stabilization by prolyl-hydroxylase inhibitor molidustat promotes the antimycobacterial activity of macrophages in Mtb infection (246). Molidustat induced the reduction of intracellular Mtb survival and upregulation of two molecules (vitamin D receptor and human beta defensin-2), which have antimycobacterial activity (246). However, the effect of HIF-1α induces different outcomes depending on macrophage activation states. Genoula et al. showed that the activation of HIF-1α induces Mtb survival by promoting foam cell formation in alternatively activated macrophages (243).

Taken together, the stabilization of HIF-1α promotes pathogen clearance in mycobacterial infection by the remodeling of metabolic pathways in macrophages, and the effect of HIF-1α can be beneficial or detrimental to the host depending on the infection stages or immune cell activation states (**Table 3**). Therefore, the role of the HIF-1α pathway in MAC infection should be for the establishment of HIF-1α-based HDT strategy for the improvement of clinical outcomes.

**Table 3:** Outcome of host-directed therapies for mycobacterial infection targeting potential pathways.

Host target	Drug	Type of study (subject)	Main finding	Outcome	References
HIF-1α	Molidustat	*In vitro* study (human peripheral blood mononuclear cells)	Hypoxia-inducible factor stabilization promotes the antimicrobial function of human macrophages	Reduced intracellular survival of Mtb	(246)
	Desferrioxamine	*In vitro* study (human monocyte-derived macrophages and alveolar macrophages)	Desferrioxamine enhances IL-1β and glycolysis in Mtb-infected human macrophages through the stabilization of HIF1α	Reduced intracellular survival of Mtb	(245)
	IFN-γ	*In vitro* study (mouse monocyte-derived macrophages)	HIF-1α is crucial for IFN-γ-mediated control of Mtb infection *in vitro* and *in vivo*	Reduced intracellular survival of Mtb	(241)
		*In vivo* study (C57BL/6 mice)		HIF-1α deficiency induces high susceptibility to Mtb infection	
	Dimethyloxalylglycine	*In vitro* study (human monocyte-derived macrophages)	Tuberculosis pleural effusion provides Mtb-favorable effects *via* metabolic shift by the inhibition of HIF-1α	Dimethyloxalylglycine protects the host from Mtb infection *via* HIF-1α stabilization	(244)
		*In vivo* study (C57BL/6 mice)			
	N/A	*In vitro* study (human monocyte-derived macrophages)	Mtb induces foam cell formation *via* the activation of HIF-1α	Promotion of the intracellular survival of Mtb	(243)
	N/A	*In vivo* study (C57BL/6 mice)	HIF-1α deficiency promotes the necrosis of *M. avium*-induced granuloma	HIF-1α deficiency induces high susceptibility to *M. avium* infection	(242)
mTOR	Everolimus	*In vitro* study (*in vitro* granuloma generated using peripheral blood mononuclear cells)	Everolimus induces autophagy *via* mTOR inhibition	Reduced intracellular survival of Mtb	(250)
		Prospective, open-label, phase 2, randomized controlled trial	Adjunctive mTOR inhibition therapy with anti-TB drugs improves the clinical outcome	Improvement of lung function than the control group	(251)
	Rapamycin	*In vitro* study (peripheral blood mononuclear cells)	mTOR inhibition during Mtb or HIV/Mtb co-infection interferes with phagosome maturation	Increased growth of Mtb during infection with interfered phagosome maturation	(252)
		*In vivo* study (C3HeB/FeJ mice)	mTOR inhibition results in host-favorable or host-deleterious outcomes according to disease stage or a combination of antibiotics	1. Adjunctive therapy of rapamycin and moxifloxacin reduced the lung immunopathology in established TB infection 2. Treatment of rapamycin alone worsened the lung immunopathology and morbidity at the early stage of TB infection	(253)
		*In vivo* study (BALB/c and C3HeB/FeJ mice)	mTOR inhibition induces beneficial or detrimental effects to the host depending on drugs	1. Treatment of rapamycin increased the bacterial burden and mortality 2. Adjunctive therapy of CC214-2 with antibiotics decreased the proportion of relapse	(254)
	Metformin	*In vitro* study (human peripheral blood mononuclear cells)	Metformin induces potentially beneficial effects on cellular metabolism, immune function, and gene transcription engaged in innate immune response against Mtb infection	1. Reduction of Mtb lysate-induced cytokine production 2. Increased phagocytosis of Mtb in macrophages	(138)
		Observational study [diabetic patients with TB (*n* = 152) and without TB (*n* = 299)]	Metformin provides a protective effect to diabetic patients with TB	1. Higher proportion of poor glycemic control in TB with diabetics 2. The protection effect of metformin was calculated as 3.9-fold	(255)
		*In vivo* study (BALB/c mice)	Metformin did not provide a host-beneficial effect to TB infection with a combination of first-line regimen	Similar lung bacterial burden and proportion of microbiological relapse	(256)
		Retrospective cohort study (patients aged ≥13 years receiving treatment for culture-positive and drug-susceptible pulmonary TB infection, *n* = 2,416)	Metformin provides beneficial effects to diabetic patients with TB	Significant reduction of mortality in metformin-treated group	(257)
		Retrospective cohort study (patients with culture-positive pulmonary TB diagnosed during 2011 and 2012)	Metformin can improve sputum culture conversion among cavitary pulmonary TB patients with diabetes	Improved culture conversion rate in cavitary pulmonary TB	(258)

N/A, Not applicable.

### mTOR pathway

Mammalian target of rapamycin (mTOR) is a serine/threonine protein kinase that orchestrates metabolism and physiological responses and is associated with human diseases such as diabetes, obesity, and cancers (259). Recent studies have focused on the regulatory role of mTOR complex 1 (mTORC1) and mTOR complex 2 (mTORC2) in innate immune response in mycobacterial infections (250–254). Ashley et al. revealed that mTOR inhibition by everolimus induces a significant reduction in Mtb burden in *in vitro* granulomas (250). In addition, everolimus showed additive effects against Mtb infection with the combination of isoniazid and pyrazinamide (250). Tasneen et al. demonstrated that the treatment of two different classes of mTOR inhibitors (rapamycin and CC214-2) provides a controversial effect in a murine TB model (254). Mice treated with rapamycin alone showed higher lung bacterial burden and mortality than the untreated controls (254). However, treatment with CC214-2 alone did not exacerbate the bacterial burden and mortality. Moreover, addition of CC214-2 to conventional antibiotic regimens significantly decreased the proportion of relapse, whereas rapamycin was not effective in suppressing a relapse (254). Similarly, Bhatt et al. reported that the treatment of rapamycin with or without moxifloxacin promotes the reduction of lung inflammation indicators such as the number and size of caseating necrotic granulomas in a C3HeB/FeJ mouse model (253). Wallis et al. reported that adjunctive mTOR inhibition therapy with anti-TB drugs improves the clinical outcome (251). Treatment of CC-11050 and everolimus did not induce an earlier culture conversion but improved the lung function at day 180 compared with the control group (251).

On the contrary, a study from Sweden showed that mTOR inhibition by rapamycin enhances Mtb growth, especially in HIV-co-infected human macrophages (252). One possible explanation for the different result from other studies is that the HIV co-infection induced the impairment of macrophage function.

Besides its role in ROS production and phagosome maturation, MET also inhibits mTORC1 signaling (260). Previous studies suggested that MET provided a beneficial effect to the host despite controversial results (138, 255–258). Lachmandas et al. demonstrated that MET induces potentially beneficial effects on cellular metabolism, immune function, and gene transcription engaged in innate immune response against Mtb infection in peripheral mononuclear cells (138). MET treatment resulted in increased Mtb phagocytosis by macrophages and the reduction of Mtb lysate-induced cytokine production (138). Clinical studies also proved the beneficial effect of MET to the host against TB infection. An observational study from India reported that MET treatment provides a 3.9-fold protective effect to diabetic patients with TB (255). Similarly, Degner et al. demonstrated that MET treatment significantly reduces mortality in diabetic patients with TB (257). Furthermore, a retrospective cohort study showed that MET improved the sputum culture conversion rate among cavitary pulmonary TB patients with diabetes (258). However, Dutta et al. suggested that MET does not improve the antimycobacterial activity of first-line anti-TB drugs in a mouse model (256). MET treatment did not reduce the lung bacterial burden or the proportion of microbiological relapse compared with a conventional anti-TB regimen (256). One possible explanation for the differing results from other studies is the species or strain of animals used (137, 141, 256). Animal studies that showed the beneficial effect of MET used C57BL/6 mice or guinea pigs for infection model, whereas Dutta et al. used BALB/c mice. Dutta et al. included rifampin in the anti-TB regimen, and rifampin may facilitate MET metabolism by activating hepatic cytochrome P450 enzymes (such as CYP2D6 and CYP3A4) (261). Activation of hepatic enzymes may reduce the concentration of MET in BALB/c mice, thus decreasing the effect of the drug.

In summary, mTOR inhibition by a wide range of drugs may provide beneficial effects to the host in mycobacterial infections based on data from clinical and experimental studies (**Table 3**). However, there is still no relevant data for the association between MAC infection and mTOR inhibition. Further studies are needed to elucidate the role of the mTOR pathway in MAC infection for the improvement of clinical outcome.

## Conclusion and future perspective

To date, investigators have explored novel therapeutic strategies for better clinical outcomes in TB. Accumulating data suggest that HDTs provide protection and aid in bacterial clearance. However, only a few studies have been conducted to identify the potential role of HDTs in MAC infection. Therefore, a deeper understanding of host–pathogen interactions is required to elucidate and validate potential HDTs—for example, recent studies have focused on diverse mechanisms that enhance intracellular survival, including foamy cell induction, modulation of autophagy and phagosome maturation, control of cell death, and manipulation of inflammasome activation in macrophages (262). Inhibition of the intracellular survival strategy of MAC by enhancing macrophage function with HDTs may be a promising therapeutic approach.

The HDTs discussed in this review are classified into two major categories—those that directly enhance macrophage function and those that modulate inflammation. Directly enhancing macrophage function is a promising strategy for bacterial clearance in the early stage of MAC lung disease. Enhancing macrophage function inhibits intracellular replication, subsequently leading to complete clearance. In contrast, modulating inflammation may provide host protection from tissue-destructive inflammation during the late stages of MAC infection. Regulation of the dynamic balance between pro- and anti-inflammatory immune responses is a critical factor for the successful control of MAC infection. This involves appropriate control of hyperactive inflammatory responses, which may lead to detrimental damage to the host. Therefore, multiple combinations of HDTs should be assessed to mediate this balance during the long-term control of MAC infection. Most of the HDTs proposed in this review have been adopted in TB treatment; therefore, we discussed the major signaling cascades reported so far in macrophages during MAC infection and selected HDTs that act at common nodes to explain their potential. Therefore, this review highlights novel host-oriented therapeutic approaches for MAC infection for better clinical outcomes, contributing to shortening the treatment duration and reducing the side effects by modulating and enhancing macrophage function.

## Author contributions

H-EP, WL, SC, MJ, M-KS, and SJS wrote the manuscript. M-KS and SJS conceived the study, supervised the team, and critically revised the manuscript. All the authors participated in the preparation and editing of the manuscript. All authors contributed to the article and approved the submitted version.

## Funding

This work was supported by Basic Science Research Program through the National Research Foundation of Korea (NRF) funded by the Ministry of Education (2021R1I1A2045131), by the NRF grant funded by the Korea Government(MSIT)(2021R1C1C2012177), and by the Bio of Medical and Medical Technologyprogram of NRF grant funded by the Korea Government(MSIT) (2020M3A9H5104234). The funders had no role in the study design, data collection and analysis, decision to publish, or preparation of the manuscript.

## Acknowledgments

The authors thank Medical Illustration & Design, a part of the Medical Research Support Services of Yonsei University College of Medicine, for all artistic support related to this work.

## Conflict of interest

The authors declare that the research was conducted in the absence of any commercial or financial relationships that could be construed as a potential conflict of interest.

## Publisher’s note

All claims expressed in this article are solely those of the authors and do not necessarily represent those of their affiliated organizations, or those of the publisher, the editors and the reviewers. Any product that may be evaluated in this article, or claim that may be made by its manufacturer, is not guaranteed or endorsed by the publisher.
